# Ulcerative Endocarditis

**Published:** 1881-07

**Authors:** G. M. Garland


### ULCERATIVE ENDOCARDITIS.

By G. M. GARLAND, M.D.

The following case is interesting on account of the great obscurity which usually surrounds ulcerative endocarditis and the consequent difficulties in the diagnosis of the same.

The patient was a very fleshy man, thirty-four years old, of sedentary habits, a liberal drinker. He never had a rheumatic fever, but a mitral regurgitant murmur has been audible over his heart for ten years. Four years ago he suffered from some spinal irritation, which lasted a few months and then disappeared. Two years ago he became jaundiced, but was comparatively well after that until February 13, 1880, when he was seized with sciatica in his left leg. He says he played a game of billiards in a warm room on the previous evening, and then stood talking on the corner of the street for some time. On February 14th he was helpless in bed, but soon improved so as to sit up and move about the room, and I made my last visit on March 1st. On the afternoon of March 4th I happened to run in for a friendly call, when I found my patient in a rocking-chair, reading, but looking terribly pale and weak. He complained of no pain, but said he was very weak, and had no appetite. His pulse was 120, rapid and feeble. The temperature was 101 5° F. I could find nothing unusual in his lungs or heart, and the sciatica was no worse, so that I was at a loss to account for his condition. On March 5th I called my friend, Dr. M. L. Chamberlin, in consultation. We found the patient in bed, with his face slightly cyanotic, and the breathing somewhat accelerated.

His wife said he had breathed rapidly for two days. He made no complaint, except of weakness. The pain in the leg was not troublesome, and with most careful examination we could find no evidence of hip-disease, and no tenderness along the spine. The pulse was 120, feeble. Temperature 101° F. Respiration 30. No pain in chest. Percussion and auscultation of the lungs normal. Mitral regurgitant murmur and hypertrophy of heart as before. Liver and spleen were enlarged. No ascites. No œdema of legs. Finding no indications but the great weakness, we ordered gin and infusion of digitalis, and requested a specimen of his urine for examination. The urine was normal. No albumen or casts.

On March 6th he was a trifle stronger, but on the following day he began to fail rapidly. Ceased to read and grew drowsy. He began to dream a great deal, and was wandering when awake. Pain in the leg was almost gone and he could move freely in bed.

On the 8th and 9th he began to have hallucinations and delusions, which were extremely pleasant in character. Also began to be aphasic, and swore roundly when misunderstood. He became slightly cross-eyed, and the eyes had a marked lateral vibratory movement. He failed to recognize me once, and then immediately apologized for it. The temperature and pulse remained the same as on previous visit.

March 11th. Brain trouble evidently increasing, but no paralysis of motion or sensation. Fails to recognize old friends, and is rambling all the time. Hypostatic congestion of lower lobe of right lung. The face is more or less cyanotic and cold to the touch, although the internal temperature is 101.5° F. Heart is working harder, as shown by heaving of chest, but no other change in that organ could be detected. Pulse 120.

March 12th. Patient has been unconscious for twelve hours. Moves freely in bed, however. Vomits all food and medicine. Speech is thick. Both sides of chest full of loud, coarse, mucous rales. No œdema of extremities. In trying to feel of feet to see if they were cold, my hand fell across the tibial artery, where I noticed a stronglymarked Corrigan pulse. The sensation to my hand was as if a tight cord were snapped up against the palm and suddenly withdrawn from the same. This pulse is pathognomonis of aortic insufficiency, and on auscultation Dr. Chamberlain and I thought we could distinguish an aortic regurgitant murmur, but the coarse mucous rales above referred to were so noisy that it was difficult to settle the question. Our confidence in the significance of Corrigan pulse was so great, however, that we diagnosed aortic valvulitis, with resulting insufficiency, as the trouble in the heart.

March 13th. Rapid superficial breathing. Profuse sweating of head. Slight increase of reflex movement on tickling feet. Scowls if face be pressed. Entirely oblivious of everybody around him. Urine passes unconsciously. No movement of bowels for four days. Nothing was retained on stomach for previous forty-eight hours. Peculiar sweet death odor noticeable on entering room. Died quietly at 4:20 P. M., that is about eleven days after beginning of cardiac attack, as nearly as can be reckoned.

The autopsy was made by Dr. W. F. Whitney on the next day, with the following result:

Autopsy twenty-four hours after death. Ice had been placed on abdomen. Body of a very large man. Rigor mortis absent. Head not opened. Subcutaneous adipose tissue largely developed. Large amount of fat deposited in omentum and mesentery. Otherwise nothing abnormal in peritoneal cavity.

Pericardium normal. Heart enlarged. Walls of left ventricle three-eighths of an inch in thickness; of right ventricle one-fourth of an inch. Color of muscle light reddish brown. Microscope showed no change in muscular structure. The free edges of the mitral valve slightly thickened and irregular, of a yellowish white color. No evidences of recent disease. The aorti valves showed insufficiency by the hydrostatic test. The free edges of the valves were irregular in outline, showing a marked loss of substance. The curtains of the valves were slightly reddened, and on the surface, especially near the edges, was a yellowish-white deposit, mixed with recently clotted blood, and on which small whiter specks were to be seen. These were found to be composed of minute round bodies (micrococci) larger than the granular detritus which composed the mass of the substance covering the valves. The right heart presented nothing abnormal.

The right lung was bound to the chest wall by moderately firm adhesions. The lung did not collapse on removal from the body, and was of a dark reddish color. Upon section a reddish fluid, mixed with bubbles of air, could be pressed from the cut surface, which was homogeneous throughout. This was especially marked in the lower lobe.

The left lung presented the same appearance, though to a less degree.

The spleen was of twice the normal size. On section the pulp was found to be of a dark reddish color, and very soft.

The kidneys were increased in size, soft. The capsule slightly adherent in places. On section the cortical substance was slightly increased in thickness, and was of a slightly opaque yellowish white color, not uniform, but giving it a streaked appearance. The outline of the separate cells, under the microscope, was not to be distinguished, and their bodies were filled with a fine granular substance.

The liver was large, of a lightish-brown color, the acini distinct from each other, but the surface had an opaque look, and was rather dry. The outline of the cells was more rounded than normal. Some of them contained well-marked fat drops, but the larger number contained fine glancing particles, not turning black with osmic acid.

Other organs presented nothing unusual.

Diagnosis: Acute ulcerated endocarditis. Œdema of lungs and hypostatic congestion. Cloudy swelling of liver and kidneys. Acute splenic tumor.

If we exclude the cyanosis, which, of course, would make one think of the heart, we notice in this case the conspicuous absence of any symptoms pointing to that organ until the destruction of the aortic valves was revealed by the Corrigan pulse. There was no præcordial pain or oppression in breathing, and the patient presented rather the appearance of typhoid fever than heart disease. There is no doubt but that this trouble is often mistaken for ague, pyæmia, enteric fever, subacute rheumatism, or tuberculosis. The beginning of the disease is always obscure. It may be ushered in by a chill or even a rigor. The pulse is then quickened, the tongue dry. Fever is a conspicuous symptom, and the temperature may rise to 104° or 102.8° F., but may also run at a lower level. The temperature often assumes a pyæmic type of irregular fluctuations, which is a very important symptom. The pulse is always rapid, 120 to 160. There may be jaundice, and articular pains are often complained of. Vomiting and diarrhœa are common.

These symptoms are general, and point in no particular direction. But now follow a series of phenomena which are very characteristic. Ulcerated endocarditis is distinguished by an accumulation of septic vegetations upon the valves of the heart. These vegetations are soon torn from their attachments, swept away, and distributed among all the organs of he body. Lodging in the skin, they produce petechiæ, and even large purpuric blotches. In the spleen they cause enlargement and softening, with tenderness to pressure. The kidneys become inflamed and swollen, and albumen appears in the urine. Emboli in the brain produce all degrees of aberration, from muttering delirium to somnolence and coma.

This series of successive explosions of nutritive and functional disturbances in numerous and remote organs, combined with great physical prostration, and possibly with pyæmic fever, are the chief symptoms which lead us to suspect this disease. Of course the case becomes immensely simplified if we obtain evidence of recent valvular changes in the heart itself. But the first point is to suspect the heart, and examine it frequently. Dr. Thompson says whenever he finds a murmur or medley of murmurs at the præcordium, combined with delirium, he thinks of embolism of the extreme arterioles of the brain, and especially in the cortical substance of the convexity.

The conditions under which this disease occur are as uncertain and ill-defined as are the symptoms which accompany it.

My case was preceded by sciatica, and the disease sometimes follows rheumatism. Dr. Osler found that out of fifty-seven cases 26.3 per cent were associated with rheumatism. The disease may occur spontaneously, and no possible cause be discovered for it, and again it may be super-imposed upon an old valvular trouble. It is occasionally secondary to some inflammatory focus. Again one case is reported where it was associated with a suppurating corn on the foot.

The nature of ulcerative endocarditis has long been the subject of discussion and experimentation. Virchow detected, many years ago, a difference between this and ordinary endocarditis. Seeking an explanation of this difference, he cut up various substances and injected them into the veins of an animal. Bits of caoutchouc simply produced the ordinary results of mechanical obstruction to the circulation, while animal substances (bits of fibrin, muscle, etc ) caused severe inflammation, suppuration and sloughing. Virchow therefore concluded that the phenomena of infection were due to chemical changes in the emboli. Others followed this idea in assuming that the irritant effects are due to the decomposition of the emboli which plug the vessels. Lanceraux thought the poisoning was caused by an alteration in the connective tissue of the valves themselves, and not by disintegration of the vegetations alone. Virchow noticed that the valvular deposits contained fine, closely-packed granules, which were insoluble in potash, acetic and hydrochloric acids, but were dissolved in chloroform. He considered them to be of a fatty nature.

Professor Winge was the first one (1869) to advance the theory that these granules are living organisms, and he called the disease mycosis endocardii. This same view was taken by Professor Heiberg, of Christiana, who sent specimens to Virchow for examination. With further study Virchow, Klebs, Koster and a number of other Germans have given their approval to this theory, and they say that the organisms found are micrococci, vibriones, bacteria and leptothrix filaments. Some of these observers believe that the germs are the essence of the disease. Heiberg inclines to the germ side of the question, but he allows that cases of ulcerative endocarditis present themselves wherein no parasites can be detected. Eberth thinks that the bacteria are introduced into the blood from without, and become aggregated into a sticky mass, which adheres to the surface of cardiac valves. Here they multiply rapidly, and, dropping off, are distributed to all parts of the body. On the other hand, some writers repudiate the germ theory with great vigor, and claim that the parasites, if present, are merely the accidents of the disease. Hiller says that bacteria and leptothrix are constantly present in the healthy mouth, and if they are so dangerous we ought to be poisoned all the time. He thinks the granules described are merely detritus.

Examining a case of ulcerative endocarditis after death, one finds one or more of the valves and portions of the endocardium covered with patches of vegetations and ulcerations. These patches are generally small in area, and may be on either side, and in any one of the four chambers of the heart. Of course, when the disease is limited to the right side of the heart, the brunt of the embolic explosion comes upon the lungs, which usually contain scattered abscesses. The liver is pale and flabby. The spleen is soft and pulpy. The kidneys present cloudy swelling, and hemorrhages may be seen in any or all of the chief organs and tissues. Dr. Hall Curtis reports a case where small spots of superficial gangrene formed on the right leg from minute emboli in the skin.

Little can be said in regard to the treatment of this formidable disease. Nearly all the cases reported have been fatal, and some were rapidly so. I have seen one doubtful case of recovery describeu, but it seems as if our chief consolation in this disease will be the satisfaction of having detected its presence before it was revealed by the autopsy.—*Boston Medical and Surgical Journal.*

